# Workshop on Emerging Technology and Data Analytics for Behavioral Health

**DOI:** 10.2196/resprot.9589

**Published:** 2018-06-20

**Authors:** David Kotz, Sarah E Lord, A James O'Malley, Luke Stark, Lisa A Marsch

**Affiliations:** ^1^ Center for Technology and Behavioral Health Geisel School of Medicine at Dartmouth Lebanon, NH United States; ^2^ Department of Computer Science Dartmouth College Hanover, NH United States; ^3^ Department of Psychiatry Geisel School of Medicine at Dartmouth Lebanon, NH United States; ^4^ Department of Pediatrics Geisel School of Medicine at Dartmouth Lebanon, NH United States; ^5^ The Dartmouth Institute for Health Policy and Clinical Practice Geisel School of Medicine at Dartmouth Lebanon, NH United States; ^6^ Department of Biomedical Data Science Geisel School of Medicine at Dartmouth Lebanon, NH United States; ^7^ Department of Sociology Dartmouth College Hanover, NH United States

**Keywords:** behavioral health, mobile technology, wearable devices, data analytics, mHealth

## Abstract

Wearable and portable digital devices can support self-monitoring for patients with chronic medical conditions, individuals seeking to reduce stress, and people seeking to modify health-related behaviors such as substance use or overeating. The resulting data may be used directly by a consumer, or shared with a clinician for treatment, a caregiver for assistance, or a health coach for support. The data can also be used by researchers to develop and evaluate just-in-time interventions that leverage mobile technology to help individuals manage their symptoms and behavior in real time and as needed. Such wearable systems have huge potential for promoting delivery of anywhere-anytime health care, improving public health, and enhancing the quality of life for many people. The Center for Technology and Behavioral Health at Dartmouth College, a P30 “Center of Excellence” supported by the National Institute on Drug Abuse at the National Institutes of Health, conducted a workshop in February 2017 on innovations in emerging technology, user-centered design, and data analytics for behavioral health, with presentations by a diverse range of experts in the field. The workshop focused on wearable and mobile technologies being used in clinical and research contexts, with an emphasis on applications in mental health, addiction, and health behavior change. In this paper, we summarize the workshop panels on mobile sensing, user experience design, statistics and machine learning, and privacy and security, and conclude with suggested research directions for this important and emerging field of applying digital approaches to behavioral health. Workshop insights yielded four key directions for future research: (1) a need for behavioral health researchers to work iteratively with experts in emerging technology and data analytics, (2) a need for research into optimal user-interface design for behavioral health technologies, (3) a need for privacy-oriented design from the beginning of a novel technology, and (4) the need to develop new analytical methods that can scale to thousands of individuals and billions of data points.

## Introduction

Digital technologies offer unprecedented opportunities to better understand and enhance behavioral health—individuals’ cognitive, emotional, and behavioral well-being. Behavioral health includes areas of substance use and mental health, and also embraces a broader spectrum of behavior (such as diet, physical activity, medical regimen adherence, and other lifestyle factors) which may impact individuals’ quality of life and health outcomes. Given the widespread access to technology worldwide, health monitoring and behavior change tools delivered on mobile platforms enable widespread reach and scalability of evidence-based interventions.

The rapidly evolving interdisciplinary field of behavioral health is making increased use of emerging technologies, novel methodologies, and data analytics in the development of effective and personalized digital therapeutic interventions. These technologies include mobile and wearable devices, and enable the delivery of personalized, “in the moment” interventions to empower patients and improve health. Novel sensing technologies allow for real-time measurement of a range of physiological, behavioral, and social activities and can help inform optimal timing of delivery of mobile interventions. Social media can be harnessed for rich data mining to develop a deeper understanding of both individual and population-level trends in health behavior and can serve as novel platforms for the delivery of behavior-change interventions. Increasingly sophisticated data analytics can be used to gain insights from mobile, sensor, and social data about individual and population health via advanced statistical methods, including machine learning and predictive modeling. Collectively, these digital technologies enable an entirely new offering of tools for collecting rich data about individuals’ behavior, health, and environment, provide personalized interventions and resources based on individuals’ needs and preferences, and enable dynamic statistical models and computational methods to predict and characterize individuals’ changing needs and health trajectories over time.

The Center for Technology and Behavioral Health (CTBH, a P30 “Center of Excellence” supported by the National Institute on Drug Abuse at the National Institutes of Health) is an interdisciplinary research center at Dartmouth College focused on the use of scientific methods to inform the optimal development, systematic evaluation, and sustainable implementation of a wide array of digital therapeutic tools for behavioral health (including web and mobile tools to help individuals manage challenges such as substance use and mental-health disorders and to promote health behavior change) [1]. In February 2017, CTBH sponsored a workshop on innovations in emerging technology, user-centered design, and data analytics for behavioral health, with presentations by a diverse range of experts in the field. Although all of the attendees and speakers were from Dartmouth, the insights from the workshop experience are broadly applicable. This paper summarizes the workshop panels on mobile sensing, user-experience design, statistics and machine learning, and privacy and security, and concludes with suggested research directions for this important and emerging field of applying digital approaches to behavioral health.

**Figure 1:**
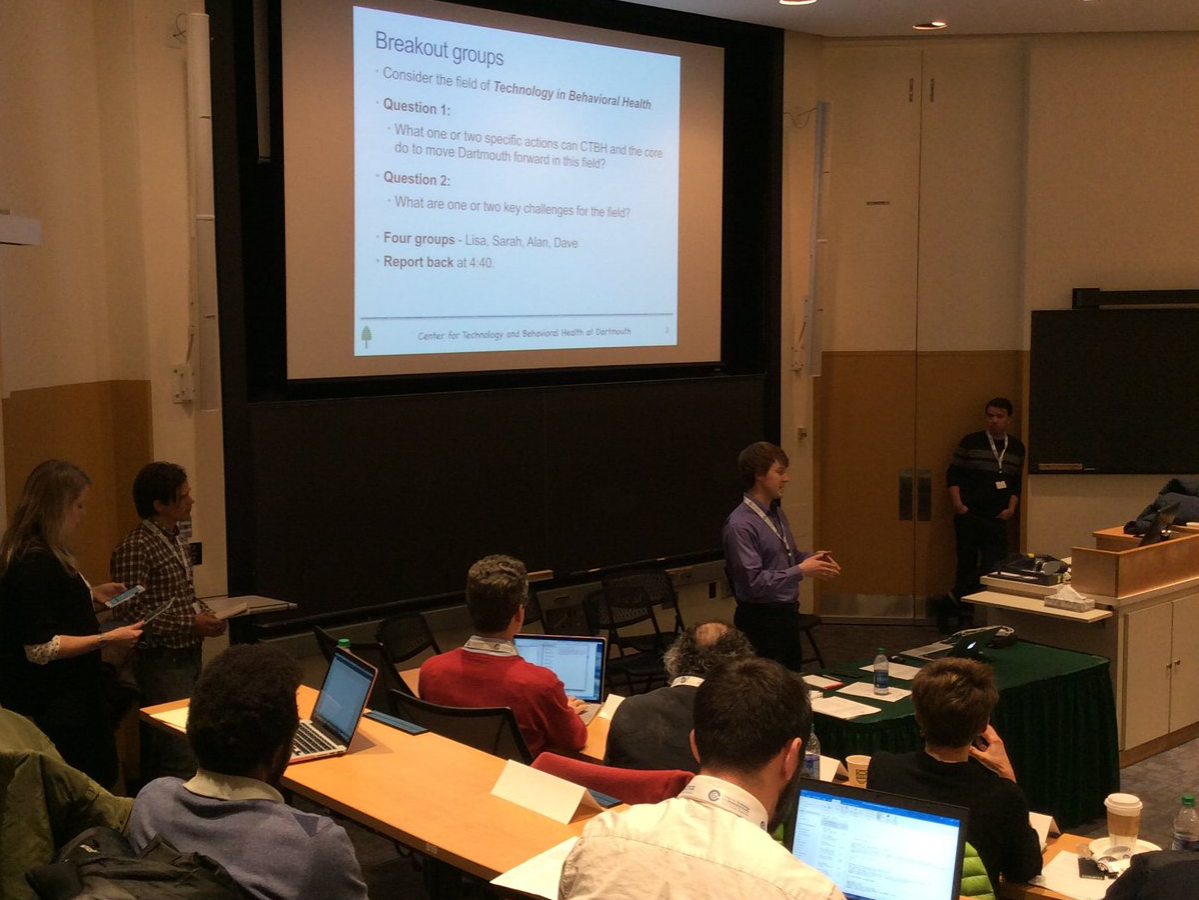
The Center for Technology and Behavioral Health (CTBH) Feb 2017 workshop.

## Mobile Sensing

*David Kotz* (Computer Science) observed that trends in health care information technology are leading to increased interest in monitoring of patients outside the clinical setting, through wearable and location-based, at-home technologies. He provided his own working definition of “mobile health”, or mHealth, as “the use of mobile computing and communications technology in the delivery of health care or collection of health information,” giving examples ranging from clinical to home use of mobile devices, to personal wellness apps, to opportunities for mHealth in the developing world.

Three other speakers described specific opportunities for mobile sensing in behavioral health. *Ryan Halter* (Engineering) described four projects underway at Dartmouth, beginning with Amulet [2,3], a collaborative effort to develop a wearable computing platform that can run multiple mHealth apps with strong security guarantees and weeks-long battery life. In a related project, Halter’s group is developing a wristband to measure electro-dermal activity which, in combination with the Amulet and a chest-strap heart-rate monitor, they use to measure stress in free-living conditions. *Kofi Odame* (Engineering) spoke about his work in ultra-low-power analog electronics, for example, in measuring wheezing, coughing, and related sounds from a wearable microphone. In a new project, Auracle [4], Halter, Odame and Kotz are developing an ear-worn device that can detect eating, in support of eating-behavior studies. They are also developing a method for bioimpedance-based cardiorespiratory monitoring, an array of electrodes worn around the chest and waist that provides real-time images of cardiac and lung function. Finally, *Bill Kelley* (Psychological and Brain Sciences) spoke about his use of smartphone sensing for a months-long study of student dysphoria, depression, and mental health in free-living conditions. They found strong correlations between the data from passive smartphone sensing with self-reported (Ecological Momentary Assessment; EMA) [5] responses and clinical instruments such as Patient Health Questionnaire-9 (a standardized measure of depressive symptoms) [6].

All the speakers expressed great optimism for the future of wearable and mobile sensing technology, bringing new insights into behavioral health and new opportunities for effective interventions, while noting significant research challenges ahead.

## User Experience Design for Behavioral Change and Health

The ease of use and aesthetic qualities of innovations, such as digital therapeutic interventions, are critical to successful dissemination and implementation of such innovations [7,8]. Presentations in this session tackled the important topics of user-interface (UI) and user-experience (UX) design for development of innovative, engaging, and effective digital approaches to behavior change and health. *Sarah Lord* (Psychiatry and Pediatrics) raised three key questions for panelists: (1) What are the best practices guiding UI/UX for digital approaches to behavioral health? (2) What are the common challenges to effective UI/UX and effective solutions to overcome those challenges? (3) How can we foster a common language among interdisciplinary teams to facilitate a successful UI/UX process for digital therapeutics for behavioral health?

*Craig Ganoe* (Data Science) provided a brief history of the field of human-computer interfaces and UI/UX, from early engineering emphasis on system performance to current iterative participatory design and development approaches. Ganoe highlighted consideration of the task-artifact cycle [9], emphasizing that assessment of UI/UX throughout the research process will yield the most effective products.

*Lorie Loeb* (Computer Science) noted that effective UI/UX promotes motivation through appeal to both emotional and intellectual aspects of individuals and presented case examples to demonstrate effective UI/UX of digital approaches for behavior change. In one case, college students worked iteratively with researchers to design and develop a simple, colorful, engaging kiosk to elicit stress assessment by college students. A 2-week pilot of the kiosk in a high traffic area of the college library yielded over 8000 responses, and positive feedback by students (personal communication by Sarah Lord, October 2017). In another case, Loeb used animation (of polar bears struggling with climate change) and data visualization (of dormitory energy-use data) and found increased energy conservation among college students [10].

*Xing-Dong Yang* (Computer Science) highlighted cutting-edge basic research focused on interaction techniques with wearable devices. Yang demonstrated an early prototype of a smartwatch face that orbits on the wristband, allowing for ready access to the watch features at any time with minimal intrusion [11]. Yang emphasized the potential of such technology for improving the lives of those with physical disabilities.

## Statistics, Machine Learning, and Data Analytics

Informatics and data analytics play an increasingly important role in understanding behavior, designing behavioral interventions, and interpreting data from behavior-related sensors. *James O’Malley* (Data Science) noted that just a few decades ago there was a sentiment among academic statisticians that “the best statisticians do not work with data,” a reflection of the focus on independent theoretical and methodological work in promotion criteria for tenure-line university-based statisticians and biostatisticians. The computing evolution has led to a reversal of priorities, with data extraction, manipulation, analysis, and presentation all deemed highly important.

Three panelists spoke about the role of mobile phone and other emerging technologies in their behavioral-health research. *Emily Scherer*, a biostatistician, described how the already-broad skillset of a biostatistician has changed due to a need to use or develop more complex analytic approaches and data gathering or acquiring techniques (eg, web-scraping) than those used in traditional randomized clinical trials and other common statistical domains or problems. For example, EMA data is an increasingly useful form of intensive longitudinal data [5]. Although EMA data is becoming commonplace due to smart devices that enable in-context monitoring of participants and adaptive interventions on patients, methods for analyzing these data are in their infancy. Scherer is leading several new lines of research, including the use of penalized functional regression modeling to identify the critical windows of time in which a patient’s mental health status is changing the most [12].

*Saeed Hassanpour* (Data Science) and *Benjamin Crosier* (Data Science) presented research characterized by large, complex, and unbalanced data sets. Hassanpour, an expert in natural-language processing, uses novel algorithms for reducing text and imaging data to forms that Crosier, an expert in quantitative social psychology, can use. They use Instagram location and network data to assess the impact of social media on a patient’s behavioral risk. They also use deep learning and convolutional neural networks to identify recurring patterns in data and classify observations or participants into groups and use other machine-learning methods to develop prediction rules often without using fully-specified statistical models.

All three—Scherer, Hassanpour, and Crosier—benefit from “Big Data” generated from enhanced monitoring capabilities, electronic medical records, high-throughput technologies, and networked research resources.

## Privacy and Security

Privacy and security are central problems for many digital apps, and as *David Kotz* (Computer Science) observed, several trends in information technology development make privacy and security concerns in health care especially acute. These trends include the need for accountable care and patient engagement, and continuous patient monitoring outside the clinical setting. Emerging threats and a changing regulatory environment further emphasize the importance of security and privacy in all digital technologies in health care settings.

mHealth technologies, enabled by the advent of mobile devices and cloud services for health-related apps, present an additional set of challenges. Given the immediate, personal impact of these devices, security and privacy concerns are even more acute. mHealth devices directly affect users’ health or health decisions, and mHealth data are inherently personal and thus highly sensitive. In addition, mHealth apps collect longitudinal data, including behavioral information, from a broad range of lifestyle activities—data which have the potential to be analyzed to produce insights about mood and personality. Perhaps most concerning, the mHealth sector encompasses a broad range of apps unbound by specific regulations protecting health data privacy. As witnessed by the recent controversy over Cambridge Analytica’s psychographic analysis of illicitly-obtained Facebook user data, these matters are increasingly matters of extreme societal concern.

Kotz, joined by *Denise Anthony* (Sociology) and *Luke Stark* (Sociology) discussed some of the central questions around privacy, security, and the broader social use of mHealth technologies, including the growing field of applications focusing on substance use and mental health. Key questions highlighted for future exploration included identifying what laws, policies, regulations and guidelines are currently in place to shape the technical developments around privacy and security in mobile health, and whether they were adequate for technical developments in the field; and what determining what kind of research was being conducted on the empirical impact of mobile health devices, particularly as related to their privacy affordances and security vulnerabilities. The three also discussed how human factors in UI/UX design, especially work on privacy-preserving or enhancing technologies and the broader tenets of privacy by design, could be part of the solution to the privacy and security concerns with mobile devices, and which laws and regulations might need modification to align with the privacy and security affordances of mobile-health technologies.

## Conclusions and Recommendations

The day-long multidisciplinary workshop left attendees feeling energized and excited about the potential for emerging technologies and data analytics to bring new insights to behavioral health, and new opportunities for innovative interventions. A few of the insights and recommendations from the presentations and conversation at the workshop are listed below.

Wearable devices bring new opportunities for sensing, computation, and interaction, with potential far beyond the capabilities of today’s fitness trackers and smartwatches. Sensor-design engineers should work closely with behavioral health scientists to understand what information would be most insightful, and seek innovative, unobtrusive mechanisms to collect that information.

Many behavioral-health studies (or interventions) use wearables, smartphones, and Web portals to interact with participants (or patients)—and yet, it remains a challenge to design interfaces that are efficient, effective, acceptable, and usable by target populations. Indeed, without careful thought to the user experience, including physical design of wearable devices and the user’s interaction with physical or digital interfaces, otherwise-innovative technologies may not be effective, or even adopted. Behavioral-health scientists and UI/UX designers should work collaboratively when developing technology-based interventions, and ideally with iterative input from representatives of the intended target end-users of the intervention.

New technologies can produce enormous amounts of data, rich with potential for insights into human behavior but require new approaches to analysis. Studies may involve hundreds, thousands, or even tens of thousands of participants—and billions of data points—requiring the application of innovative approaches in statistical methods, machine learning, and data analytics. Health researchers and data scientists should collaborate to bring new approaches to these rich new data sources.

Privacy should be a key factor in the design of all behavioral-health technologies, from conceptualization through prototyping and potential commercialization. Numerous design principles and heuristics, most notably those of privacy by design, are available as resources in these endeavors. Consistent with these design principles, designers and researchers should seek effective means to communicate to the end-user about what data is being collected, how it will be used, who will have access, and how it can be disposed. Poor attention to privacy may impede adoption, especially in a global regulatory context, now including the European Union’s General Data Protection Regulation, whereas strong privacy protections can encourage adoption among sensitive populations.

Mobile technologies can collect a wide range of data about individuals, including types of data not traditionally considered to be protected health information or personally identifiable information, but which (with modern analytic methods) can extract insights about sensitive behaviors and even reidentify anonymous individuals. Technology developers should limit data collection to only that which is needed, avoid transmitting and storing raw sensor data, and put strong technical and administrative controls on secondary use of data.

The Center for Technology and Behavioral Health at Dartmouth, as a community of researchers from sensor engineering, computer science, data analytics, psychiatry, and behavioral health, is poised to pursue these opportunities, and encourages researchers everywhere to engage with these exciting challenges.

## Abbreviations

CTBH: Center for Technology and Behavioral Health
EMA: ecological momentary assessment
mHealth: mobile health
UI: user interface design
UX: user experience
